# Comparative Analysis of Classification Criteria in IgG4-Related Disease and Evaluating Diagnostic Accuracy from a Retrospective Cohort in Clinical Practice

**DOI:** 10.3390/diagnostics14222583

**Published:** 2024-11-17

**Authors:** Marta Lopez-Gomez, Patricia Moya-Alvarado, Hye Sang Park, Mar Concepción Martín, Sara Calleja, Helena Codes-Mendez, Berta Magallares, Iván Castellví, Antonio J. Barros-Membrilla, Ana Laiz, César Diaz-Torné, Luis Sainz, Julia Bernárdez, Laura Martínez-Martinez, Hèctor Corominas

**Affiliations:** 1 Rheumatology Department, Hospital Universitario Araba, 01009 Vitoria, Spain; 2 Instituto de Investigación Biomédica BIORABA, Hospital Universitario Araba, 01009 Vitoria, Spain; 3 Department of Medicine, Universitat Autònoma de Barcelona (UAB), 08193 Barcelona, Spain; hcodes@santpau.cat (H.C.-M.); berpauma@hotmail.com (B.M.); icastellvi@santpau.cat (I.C.); hcorominas@santpau.cat (H.C.); 4 Institut de Recerca Sant Pau (IR SANT PAU), 08041 Barcelona, Spain; hsang@santpau.cat (H.S.P.); alaiz@santpau.cat (A.L.); cdiazt@santpau.cat (C.D.-T.); lsainz@santpau.cat (L.S.); 5 Rheumatology Department, Hospital de la Santa Creu i Sant Pau, 08025 Barcelona, Spain; juliabernardez8@gmail.com; 6 Gastroenterology Department, Hospital de la Santa Creu i Sant Pau, 08025 Barcelona, Spain; mconcepcion@santpau.cat; 7 Immunology Department, Hospital de la Santa Creu i Sant Pau, IIB Sant Pau, UAB, 08025 Barcelona, Spain; scallejavara@gmail.com (S.C.); lmartinezma@santpau.cat (L.M.-M.); 8 Cardiology Department, Hospital de la Santa Creu i Sant Pau, 08025 Barcelona, Spain; abarros@santpau.cat

**Keywords:** IgG4, diagnostic criteria, ACR/EULAR

## Abstract

Introduction: We conducted a comprehensive comparative analysis of the Okazaki, Umehara, and American College of Rheumatology/European League Against Rheumatism (ACR/EULAR) classification criteria for diagnosing immunoglobulin G4-related disease (IgG4-RD). Materials and Methods: A retrospective study was conducted in a single tertiary hospital, using expert clinical judgment as the gold standard. We compared the diagnostic accuracy of the Okazaki, Umehara, and ACR/EULAR criteria in a cohort of 41 patients with suspected IgG4-RD. We assessed sensitivity, specificity, and positive and negative predictive values for each criterion, and conducted a separate analysis based on four IgG4-RD subtypes. Results: A total of 30 patients were confirmed to have IgG4-RD and 11 were identified as mimickers. The Umehara criteria demonstrated the highest sensitivity (83.33%), followed by the ACR/EULAR 2019 (66.67%) and Okazaki (60.0%) criteria. All three criteria exhibited 100% specificity, with overall diagnostic accuracy ranging from 70% to 88%. The areas under the curve (AUC) were 0.917 (Umehara), 0.800 (Okazaki), and 0.833 (ACR/EULAR 2019), indicating significant diagnostic effectiveness (*p* < 0.000). Subtype analysis revealed that the Umehara and ACR/EULAR 2019 criteria were more effective in diagnosing pancreato-hepato-biliary involvement (subtype 1), while the Okazaki and ACR/EULAR 2019 criteria were more effective in diagnosing retroperitoneal fibrosis and/or aortitis (subtype 2). Conclusions: Our study provides valuable insights into the diagnostic performance of the Okazaki, Umehara, and ACR/EULAR criteria for a cohort of patients with suspected IgG4-RD. The Umehara criterion demonstrated the highest sensitivity, suggesting its potential utility for screening purposes, while all three criteria showed consistent specificity.

## 1. Introduction

Immunoglobulin G4-related disease (IgG4-RD) is an immune-mediated chronic disorder characterized by intense inflammation, fibrosis, infiltration, and elevated IgG4 levels in peripheral blood affecting multiple organs [1,2,3]. This fibroinflammatory condition is characterized by an initial inflammatory phase driven by antigen-experienced lymphocytes secreting pro-fibrotic molecules, followed by a fibrotic phase marked by dense stromal reactions and leading to tissue distortion and organ damage.

While IgG4 antibodies normally resolve inflammation, in IgG4-RD, they may synergize with cytotoxic T cells to induce tissue injury. The antigens triggering the disease and the reasons for organ specificity remain unclear but suggest a breach of immunological tolerance as the potential initiating event [4]. IgG4-RD poses diagnostic challenges as it is often confused with neoplasms, infections, or other immunological conditions due to shared clinical, serological, and pathological features [5].

Although the prevalence of IgG4-RD in Europe has not specifically been described to date, it is estimated to be around 4.6 per 100,000 individuals, with a higher incidence among persons aged over 60 [6]. A multicenter observational study by Fernandez et al. [7] identified 55 Spanish patients with IgG4-RD, making this the largest series reported in Spain and contributing to an understanding of the disease. Singly [1] published estimates suggesting that the average time from symptom onset to disease diagnosis is two years.

Despite growing interest and scientific advances, diagnosing IgG4-RD remains a significant challenge for clinicians due to its complexity and heterogeneity. Moreover, IgG4-RD requires an integrated approach, involving comprehensive evaluation and proper coordination among different medical specialties.

The organs most frequently affected are the pancreas, retroperitoneum, salivary glands, and lymph nodes [7]. The average number of affected organs is 2.9 and isolated involvement of a single organ is very uncommon [4].

The IgG4-RD predilection for certain organs has been known since early on, although comprehensive assessments of consistent patterns in clinical manifestations were lacking until 2019, when, applying latent class analysis to an international cohort, the American College of Rheumatology (ACR)/European League against Rheumatism (EULAR) developed classification criteria for four homogeneous phenotypes of IgG4-RD, as follows: pancreato-hepato-biliary disease (31%), retroperitoneal fibrosis and/or aortitis (24%), head/neck-limited disease (22%), and Mikulicz syndrome with systemic involvement (22%) [1]. Notably, patients within each phenotype shared distinctive clinical, epidemiological, and serological features. Patients with head/neck limited disease were more likely to be female, Asian, and younger, and more frequently required histological confirmation for diagnosis. Patients with pancreato-hepatobiliary disease were more often referred to the emergency department due to symptom flare-ups. In the retroperitoneal fibrosis and Mikulicz syndrome patients, inflammatory markers were significantly higher and lower, respectively. Finally, patients with Mikulicz syndrome and systemic involvement had the highest median serum IgG4 concentrations [1]. This classification provides a framework for recognizing IgG4-RD phenotypes, enabling the description of biological differences, the assessment of biomarker performance in homogeneous cohorts, and the search for personalized follow-up and therapeutic strategies.

While no genetic risk factors have been definitively linked to gender differences in IgG4-RD, environmental factors like smoking and occupational exposures are more frequently observed in male patients, potentially contributing to the higher prevalence of the disease in men [8,9,10,11].

Definitive diagnosis requires rigorous clinical–pathological correlation, as clinical, laboratory, and imaging findings are often non-specific [12]. Serum IgG4 elevation (55–97% of cases) correlates with organ involvement but is of poor diagnostic utility [4,12]. The absence of disease-specific autoantibodies (e.g., ANCA, SSA/Ro, SSB/La, double-stranded DNA, RNP, and Sm), helps differentiate IgG4-RD from other autoimmune conditions [3]. Histological examination remains the diagnostic mainstay but can be challenging for certain organs (such as the retroperitoneum and ocular cavity). Whole-body computed tomography (CT) or fludeoxyglucose F18 (18FDG)-positron emission tomography (PET) scans are recommended at diagnosis for staging and to identify accessible biopsy sites [13].

For the above reasons, classification criteria have been established based on joint evaluation of clinical, serological, and histological findings. The Okazaki criteria [14] (Appendix A), introduced in 2009, were the first to establish a standardized approach to IgG4-RD diagnosis. These criteria rely on a combination of clinical features, serological findings, and histological evidence. While their simplicity and high specificity make them suitable for initial screening, their low sensitivity requires further evaluation. The Umehara criteria [15] (Appendix B), proposed in 2012, expanded on the Okazaki criteria by incorporating additional clinical and serological parameters. They categorize cases into possible, probable, or definitive IgG4-RD. While this classification system has enhanced sensitivity compared to the Okazaki criteria, its complexity and lower specificity warrant cautious interpretation. Globally, despite clear clinical suspicion indicating the presence of the condition, neither of those diagnostic criteria are optimal, making accurate diagnosis and timely intervention not only challenging, but also underscores their inadequacy in terms of capturing the full spectrum of conditions and manifestations.

To adequately enhance the ability to identify and manage patients with IgG4-RD, the development of new criteria became imperative. In 2019, ACR/EULAR experts introduced novel classification criteria for IgG4-RD [16] (Appendix C), based on a three-stage approach that integrates clinical findings, serological data, and radiological findings. While the sensitivity (82–85%) of the ACR/EULAR 2019 criteria is the highest of the three sets of criteria, its complexity requires careful consideration. One notable advantage of the ACR/EULAR 2019 criteria is the ability to classify patients as having IgG4-RD even when obtaining a biopsy is challenging and/or serum IgG4 levels fall within the normal range despite clinical suspicion of IgG4-RD. Additionally, the ACR/EULAR 2019 criteria streamline the identification of homogeneous IgG4-RD cases in clinical trials, improving the availability and implementation of criteria in research settings.

While existing classification systems have individually advanced IgG4-RD diagnosis, a comprehensive comparative analysis of their clinical performance remains unexplored. As a rare, relatively novel, and often underdiagnosed condition due to low clinical suspicion and complex and unfamiliar classification criteria, IgG4-RD necessitates a thorough evaluation of existing diagnostic frameworks.

This study aims to address this critical knowledge gap by conducting a comparative analysis of the diagnostic performance (sensitivity, specificity, and accuracy) of the Okazaki, Umehara, and ACR/EULAR 2019 criteria for a cohort of confirmed IgG4-RD patients. The rationale for this research is multifaceted: (1) to optimize the diagnostic approach and facilitate timely and accurate diagnoses, crucial for appropriate management and prevention of organ damage; (2) to provide evidence-based guidance to clinicians on the most appropriate criteria for various clinical contexts, thereby potentially improving diagnostic accuracy and patient outcomes; and (3) to advance IgG4-RD research by identifying the most robust classification criteria, thereby enhancing the homogeneity of patient cohorts in clinical trials and observational studies, leading to more reliable and generalizable research findings. By offering a clearer understanding of the strengths and limitations of each set of criteria, we seek to contribute to more accurate diagnoses, more effective treatments, and better outcomes for patients with this challenging condition. Our comparative analysis is anticipated to offer valuable insights into the relative strengths and limitations of each set of criteria and provide a nuanced understanding of their applicability across diverse clinical scenarios. Ultimately, our study strives to fill a crucial knowledge gap, inform clinical practice, guide future research, and accelerate scientific progress regarding IgG4-RD, with the overarching goal of improving care for patients affected by this complex disorder.

## 2. Materials and Methods

### 2.1. Study Design and Setting

A retrospective cross-sectional study was conducted at the rheumatology outpatient clinic of a tertiary hospital designated as a Spanish national reference center for systemic autoimmune diseases (XUEC CSUR, Hospital Sant Pau i de la Santa Creu, Barcelona, Spain). The study was approved by the Hospital Sant Pau i de la Santa Creu ethics committee following the good clinical practices protocol (IIBSP-EIG-2022-92) and was performed in accordance with the tenets of the 1964 Helsinki Declaration and its subsequent amendments.

### 2.2. Patients

All patients aged 18 years and older with suspected IgG4-RD based on clinical grounds and elevated IgG4 levels (>135 mg/dL) were included from January 2000 to January 2024. The patients were evaluated by experienced rheumatologists and categorized into two groups based on expert clinical judgment: those diagnosed as having and not having IgG4-RD (IgG4-RD group and non-IgG4-RD group, respectively).

### 2.3. Procedures

Obtained retrospectively from the hospital’s electronic healthcare records were patient demographic data (sex, age, race) and disease-related variables, including clinical and laboratory parameters, histological findings, previous neoplasia, cause of death (any cause), and prescribed treatments.

The following specific clinical manifestations were also recorded: glandular involvement, pulmonary involvement, retroperitoneal fibrosis, autoimmune pancreatitis, renal involvement, aortitis, inflammatory aortic aneurysm, lower back pain, constitutional symptoms, fever, hypophysitis, pachymeningitis, orbital pseudotumor, and ocular involvement. Visceral involvement was assessed through imaging techniques including CT, PET, and/or nuclear magnetic resonance imaging (nMRI).

Recorded laboratory data included hemogram; total IgG and IgG4, C-reactive protein (CRP), erythrocyte sedimentation rate (ESR), and tests results for the presence or absence of anti-extractable nuclear antigens (ENA), rheumatoid factor, antinuclear antibodies (ANA), and various disease-specific autoantibodies (including anti-Ro/SSA, anti-La/SSB, anti-neutrophil cytoplasmic, anti-double stranded DNA, anti-RNP, anti-Sm, and plasmablasts). IgG4 quantification was performed from peripheral blood samples by turbidimetry using the Optilite kit. Reference values at our hospital were 3.9–86 mg/dL, though the upper limit of normal serum IgG4 concentration was set to 135 mg/dL, a threshold commonly used in Japanese diagnostic criteria for IgG4-RD [13,14]. Plasmablasts were identified through flow cytometry based on the expression of surface proteins (CD19 + IgM − IgD − CD27 + CD38^high^).

The three classification sets of criteria (Okazaki: present or absent; Umehara: possible, probable, definitive; ACR/EULAR 2019: present or absent and quantitative according to a weighted numeric score) were evaluated across the entire patient cohort and compared for the four IgG4-RD subtypes (pancreatic-hepato-biliary disease; retroperitoneal fibrosis and/or aortitis; head/neck-limited disease, and Mikulicz syndrome with systemic involvement) [1].

Finally, histopathological confirmation, where available, was obtained for patients in both the IgG4-RD and non-IgG4-RD groups. However, in some cases, particularly those with deep-seated fibrotic disease such as retroperitoneal fibrosis, histological confirmation was not possible due to the invasive nature of biopsy procedures and the associated clinical risks, including bleeding and vascular complications. The presence of the following histopathological features was also recorded: lymphoplasmacytic infiltration, obliterative phlebitis, and/or glandular fibrosis.

### 2.4. Statistical Analysis

Patient demographic, clinical, and biochemical data were summarized using percentages, means, and standard deviations (SD), or medians and interquartile ranges (IQR), depending on the characteristics of the variable. Differences in values of continuous variables between IgG4-RD and non-IgG4-RD groups were analyzed by Student’s *t*-test or the Mann–Whitney U test. Categorical variables were compared between groups using chi-square or Fisher’s exact tests. IgG4 subtypes in terms of either demographic or laboratory values were compared by analysis of variance (ANOVA) for continuous variables and the chi-square test for categorical variables.

Sensitivity, specificity, positive predictive value (PPV), and negative predictive value (PPV) were calculated for the Okazaki, Umehara, and ACR/EULAR 2019 criteria (note that, for the sensitivity analysis of the Umehara criteria, the possible, probable, and definitive categories were either analyzed separately or collapsed into a single disease-positive category). Values were summarized using contingency tables for true positive (T+), false positive (F+), true negative (T−), and false negative (F−) values to obtain full information on sensitivity, specificity, and diagnostic odds ratios. A receiver operator characteristics (ROC) curve was plotted, and the area under the ROC curve (AUC) was calculated to evaluate the IgG4-RD diagnostic performance of each set of criteria.

All analyses were conducted for a significance level of 0.05. Analyses were performed using R v. 4.3.1 (R Core Team (2022). R: A language and environment for statistical computing. R Foundation for Statistical Computing, Vienna, Austria).

## 3. Results

### 3.1. General IgG4-RD Characteristics

Of the 41 patients with suspected IgG4-RD included in the study, 30 cases were confirmed as IgG4-RD based on expert clinical judgment and the remaining 11 cases were classified as non-IgG4-RD. The mean (SD) age at the time of IgG4-RD diagnosis was 61.79 (16.27).

Baseline characteristics for the IgG4-RD group and non-IgG4-RD group are described in Table 1. The definitive diagnoses of the 11 non-IgG4-RD patients were retroperitoneal fibrosis (*n* = 5), inflammatory aortic aneurysm (*n* = 3), autoimmune pancreatitis (*n* = 2), and hypophysitis (*n* = 1).

Comparative analysis of the IgG4-RD (*n* = 30) and non-IgG4-RD (*n* = 11) groups revealed several significant differences. In the IgG4-RD group, males were more prevalent (83.3% vs. 36.3%; *p* = 0.007), median serum IgG4 levels were higher (90.5 vs. 47.0; *p* = 0.05), and the median plasmablast count in peripheral blood was significantly more elevated (993.0 vs. 24; *p* = 0.004). These findings highlight the utility of elevated IgG4 as a biomarker for diagnosing IgG4-RD and underscore the importance of integrating both clinical characteristics with serological biomarkers for accurate diagnosis.

All 30 patients in the IgG4-RD group had negative results for rheumatoid factor, ANA, and ENA.

Histological data are summarized in Table 2. Samples were obtained from nine patients in the IgG4-RD group, compared to just one patient in the non-IgG4-RD group (34.6% vs. 9.09%; *p* = 1.000). A notable limitation of this study is the lack of histological confirmation for most of the non-IgG4-RD patients. This was primarily due to the clinical presentation of these subjects, who exhibited retroperitoneal fibrosis associated with aortic aneurysm and mildly elevated serum IgG4 levels. The potential risks of retroperitoneal biopsies in such cases, including bleeding and vascular complications, were deemed to outweigh the diagnostic benefits.

While no patients in the non-IgG4-RD group received intravenous methylprednisolone (IV-MTPD) boluses or steroid therapy, all patients in the IgG4-RD group underwent treatment with these interventions. This stark contrast in treatment approaches highlights a key difference in the management of the two patient cohorts. Two patients (6.7%) in the IgG4-RD group who received IV-MTPD boluses followed by oral prednisone (median dose (IQR) 55 (30–60) mg) as initial treatment showed a statistically significant difference (*p* < 0.001) compared to the non-IgG4-RD group. Additionally, four patients (13.3%) required treatment with rituximab (500 mg), administered in two infusions separated by 15 days and a maintenance dose over two weeks every six months.

### 3.2. Okazaki, Umehara, and ACR/EULAR 2019 Criteria

We evaluated these criteria in a cohort of 41 patients, including 30 with confirmed IgG4-RD and 11 without IgG4-RD.

Of the 30 patients diagnosed with IgG4-RD, 18 (60%) met the Okazaki criteria; 25 (83.33%) met the Umehara criteria, including 6 (20%) definitive, 2 (6.7%) probable, and 17 (56.7%) possible cases; and 20 (66.7%) met the ACR/EULAR 2019 criteria, with a median (IQR) value of 20.0 (20–44).

Table 3 summarizes the diagnostic performance of the Okazaki, Umehara, and ACR/EULAR 2019 criteria.

For the 30 IgG4-RD patients, sensitivity was highest for the Umehara criteria (83.33%; 95% CI: 65.28–94.36%), which correctly identified 25 patients, followed by the ACR/EULAR 2019 criteria (66.67%; 95% CI: 47.19–82.71%), identifying 20 patients, and the Okazaki criteria (60%; 95% CI: 40.6–77.34%), identifying 18 patients. Further stratification of patients according to the Umehara criteria into possible, probable, or definitive IgG4-RD, the sensitivity values were 56.67% (95% CI: 37.43–74.54%), 6.67% (95% CI: 0.82–22.07%), and 20% (95% CI: 7.71–38.57%), respectively.

Specificity for all three criteria was 100% (95% CI: 71.51–100%), with all 11 non-IgG4-RD patients correctly identified, and resulting in a PPV of 100% for each set of criteria.

Accuracy was highest for the Umehara criteria, which correctly classified 87.8% (95% CI: 73.8–95.92%) of the patients, followed by the ACR/EULAR 2019 criteria (75.61%; 95% CI: 59.7–87.64%) and the Okazaki criteria (70.73%; 95% CI: 54.46–83.87%).

The NPV was highest for the Umehara criteria at 68.75% (95% CI: 41.34–88.98%), followed by the ACR/EULAR 2019 criteria, at 52.38% (95% CI: 29.78–74.29%), and the Okazaki criteria, at 47.83% (95% CI: 26.82–69.41%).

**Figure 1:**
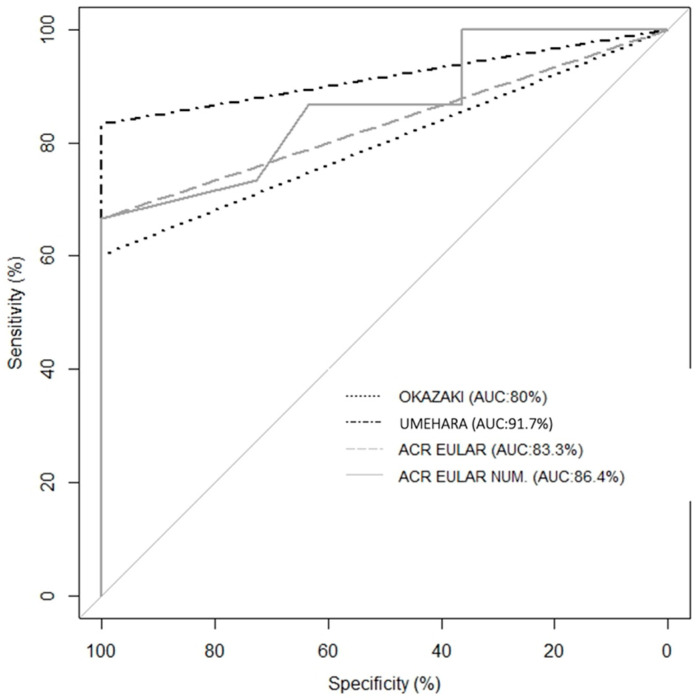
Area under the curve for the Okazaki, Umehara, and ACR/EULAR criteria.

The AUC values (Figure 1) were 0.917 for the Umehara criteria, 0.800 for the Okazaki criteria, and 0.833 and 0.864 for the ACR/EULAR 2019 criteria and numeric score, respectively, with all values showing significant diagnostic performance (*p* < 0.000).

### 3.3. Description of IgG4-RD Subtypes

The analysis of IgG4-RD subtypes revealed distinct patterns across four identified groups (Table 4). Subtype 1, comprising 26.7% of cases, was characterized by a strong male predominance (87.5%) and the most pronounced pancreatic involvement, with 75% of cases showing pancreatic–biliary injury. Subtype 2 was the most prevalent subtype, comprising 19 patients (63.3%). Was especially associated with hypertension (*p* = 0.066), aneurysms (47.4%) and retroperitoneal involvement. Pulmonary manifestations, specifically peribronchovascular and septal thickening, were exclusive to this subtype (26.3% of cases). Subtype 3 (head/neck-limited) was represented by a single case, limiting comparative analysis. Subtype 4 (Mikulicz-systemic involvement), with only two cases, showed a tendency for systemic manifestations, including aneurysm in one case.

On comparing diagnostic criteria across groups, distinctive trends emerged. The diagnostic accuracies of Umehara’s criteria and the ACR/EULAR 2019 criteria for subtype 1 were 50% and 75%, respectively. For subtype 2, ACR/EULAR 2019 demonstrated an accuracy of 68.4%, while the Okazaki’s criteria exhibited an accuracy of 63.2%. These findings suggest that specific criteria may be more useful for the diagnosis of these particular subtypes. Finally, only the subtype 3 case met both Okazaki’s and Umehara’s criteria, while the two subtype 4 cases met Umehara’s criteria.

## 4. Discussion

We evaluated the diagnostic performance of the Okazaki, Umehara, and ACR/EULAR 2019 IgG4-RD classification criteria for a cohort of 41 Spanish patients with suspected IgG4-RD, in 30 of whom the diagnosis was confirmed by expert clinical judgment. Sensitivity was highest for the Umehara criteria (83.3%), followed by the ACR/EULAR 2019 criteria (66.66%) and the Okazaki criteria (60.0%).

Regarding the Okazaki (2009) and Umehara (2011) criteria, sensitivity was suboptimal for our cohort, which would question their utility for IgG4-RD screening in clinical practice. For the ACR/EULAR 2019 criteria, our sensitivity value of 66.66% was lower than reported (85.5% and 82.0%) in the original validation studies [15]. This discrepancy can be attributed to several factors. Firstly, the inherent design of the ACR/EULAR 2019 criteria gives them a built-in advantage, as the pre-defined threshold values for sensitivity and specificity during the criteria development process effectively established a high pre-test probability. This approach inherently increased the likelihood of achieving superior diagnostic performance in the initial validation cohort. Secondly, our relatively small sample may have influenced our results, while potential differences between our cohort and the original validation cohorts (disease phenotypes, patient characteristics, geographic variations) may contribute to the observed differences. Consequently, the ACR/EULAR 2019 criteria may not achieve the same level of sensitivity when applied to different patient populations or in real-world clinical settings, as in our study. This underscores the importance of external validation studies in diverse populations to assess the true performance of diagnostic criteria outside their development cohort and to account for variations in patient demographics and clinical presentations across different settings.

Notably, in our cohort all three criteria demonstrated excellent specificity (100%) and PPV (100%). These findings align with previous studies highlighting the high specificity of these diagnostic criteria for IgG4-RD [13,14,15].

The Umehara criteria, when stratified into possible, probable, or definitive IgG4-RD categories, showed lower sensitivity than when stratified dichotomously (with/without IgG4-RD). This observation aligns with Carruthers et al. [11], who reported significantly reduced sensitivity (36%) when considering only the definitive category. These findings underscore the heterogeneous nature of IgG4-RD and the challenges associated with strict compliance with more stringent classification criteria in specific cases. To optimize diagnostic sensitivity and prevent the omission of true cases, our study highlights the importance of considering all classification criteria categories, rather than focusing solely on the category with the highest specificity.

Subgroup analysis revealed differing compliance patterns for the four IgG4-RD phenotypes (classified according to the Wallace et al. 2019 [1] categorization), suggesting the potential utility of applying specific criteria to identify certain disease subtypes.

In our cohort, retroperitoneal fibrosis and/or aortitis (subtype 2) was the most prevalent subtype, comprising 63.3% of our patients. This differs from the literature, which reports pancreato-hepato-biliary involvement (subtype 1) to be the most prevalent subtype, accounting for 31% of cases [1]. The greater prevalence of subtype 2 in our cohort can be attributed to the presence of a multidisciplinary specialized aorta monthly committee in our institution, which likely led to increased detection and referral of cases with retroperitoneal fibrosis and aortitis. The varying prevalence of subtypes in our cohort, along with differences in how well each subtype meets different diagnostic criteria, underscores the known heterogeneity of IgG4-related disease and highlights the challenges faced in creating universally applicable diagnostic criteria.

Aligning with previous reports advocating for customized diagnostic approaches based on phenotype-specific consideration [1,14,15], our findings underscore the importance of considering disease phenotypes when selecting appropriate diagnostic criteria. We found that the Umehara and ACR/EULAR 2019 criteria performed better for the pancreato-hepato-biliary subtype, whereas the Okazaki and ACR/EULAR 2019 criteria performed better for the retroperitoneal fibrosis and/or aortitis subtype [17]. Adams et al. [5] also found that the ACR/EULAR 2019 criteria were more effective for both pancreato-hepato-biliary and retroperitoneal fibrosis and/or aortitis subtypes, corroborating Martinez-Calabuig et al. [18], who found that the ACR/EULAR 2019 criteria were superior to the Umehara and Okazaki criteria in diagnosing these subtypes. Those findings overall suggest that the ACR/EULAR 2019 criteria can play a crucial role in enhancing diagnostic accuracy for the IgG4-RD pancreato-hepato-biliary and retroperitoneal fibrosis and/or aortitis subtypes, thereby potentially improving patient care and outcomes.

The mean age of IgG4-RD diagnosis in our cohort was 61.79 years, which aligns with Brito-Zerón et al.‘s [19] observation that the disease predominantly affects older adults.

Likewise, men predominated among our patients with an IgG4-RD diagnosis, highlighting potential sex-specific differences in disease presentation and susceptibility. Although no definitive disparity hypothesis is supported, men with IgG4-RD typically exhibit more a frequent involvement of organs such as the pancreas and kidneys, accompanied by a more active B-cell response [6].

The main strength of our study is the novelty of simultaneously comparing all three sets of criteria (Okazaki, Umehara, and ACR/EULAR 2019) in a cohort of IgG4-RD patients which, to our knowledge, is the first such comparison to date. While previous studies have compared the ACR/EULAR 2019 and the Umehara criteria separately, no single study has comprehensively evaluated the diagnostic performance of all three criteria sets concurrently. Additionally, our cohort includes patients representing all four IgG4-RD phenotypes, although, importantly, there was a significant imbalance in their distribution, in contrast with the leading ACR/EULAR groups [1]. In our cohort, subtypes 2 and 1 were predominant, while subtypes 3 and 4 were only anecdotally represented. This disproportionate distribution makes it challenging to draw robust conclusions regarding the latter two groups. Consequently, while our study provides some insights into the potential utility of specific criteria for different subtypes, the reliability of these insights varies considerably across subtypes.

The strict selection of patients with a high pretest probability of IgG4-RD (based on stringent inclusion criteria and comprehensive evaluation) likely contributed to the observed 100% PPV across all three criteria and minimized the inclusion of false negatives, while meeting the classification criteria significantly increased the likelihood of true positives. These findings highlight the clinical utility and reliability of the diagnostic criteria in accurately identifying IgG4-RD cases within our study cohort.

The main limitations are the small sample size, reflecting the low prevalence of the disease, and the single-center nature of our study. Further studies with larger sample sizes are necessary to achieve greater robustness. We plan to include all potential cases of IgG4-RD evaluated in our hospital for a future multicenter study to validate our results. Another key limitation was that enrolled patients were selected based on high clinical suspicion of IgG4-related disease and elevated IgG4 levels. This selection process may not have captured the full clinical spectrum of the condition, as patients presenting with subtle or atypical manifestations, or those with normal IgG4 levels despite active disease, could have been inadvertently excluded. To help mitigate this potential selection bias, the final diagnosis was determined through comprehensive evaluation by physicians with specialized expertise in IgG4-RD. However, this approach does not entirely eliminate the possibility that the study population may not be fully representative of the broader IgG4-RD patient population.

Additionally, since histopathological confirmation is crucial to diagnosing IgG4-RD in a non-negligible proportion of cases, a significant limitation was the small number of biopsies performed. Consequently, we may have inadvertently excluded a subset of patients with the disease. The paucity of biopsies in our cohort was due to the predominance of IgG4-RD subtype 2 (retroperitoneal fibrosis and aortitis). As reported in the literature, retroperitoneal fibrosis often presents with a fibrotic phenotype, meaning that tissue sampling is not only technically difficult (due to the deep-seated nature and proximity to vital structures) but may also yield limited diagnostic information (prominent fibrosis but minimal inflammation) [20]. This makes diagnosis particularly challenging, especially in the absence of distinctive histopathological features. Therefore, despite the recognized importance of biopsy for definitive diagnosis, the anatomical and pathological characteristics of subtype 2 often render biopsies unfeasible or potentially hazardous, further complicating the diagnostic process. Consequently, while our study provides some insights into the potential utility of specific criteria for different subtypes, the reliability of these insights varies considerably across the subgroups due to their uneven representation.

## 5. Conclusions

This retrospective study provides a detailed comparative evaluation of the Okazaki, Umehara, and ACR/EULAR 2019 classification criteria for diagnosing IgG4-related disease (IgG4-RD) in a Spanish cohort. Although the overall sample size is small, our findings demonstrate that the Umehara criteria exhibit the highest sensitivity (83.33%), making them the most suitable for screening purposes. Meanwhile, all three sets of criteria showed perfect specificity and positive predictive value (PPV), effectively confirming the diagnosis of IgG4-RD.

The predominance of subtype 2 in our cohort, attributed to a specialized multidisciplinary approach at our center, further supports the importance of clinical context in the diagnosis of IgG4-RD. However, the uneven distribution of subtypes, particularly the underrepresentation of subtypes 3 and 4, limits the generalizability of our conclusions for these less prevalent subtypes.

The lack of histopathological confirmation in some cases, particularly in patients with deep-seated fibrotic disease, remains a limitation, emphasizing the need for alternative diagnostic strategies when biopsy is not feasible. Future multicenter studies with larger, more balanced cohorts and broader histopathological validation are needed to refine these diagnostic criteria and improve their applicability across different IgG4-RD phenotypes.

In summary, our results suggest that while no single set of criteria is universally optimal, the Umehara and ACR/EULAR 2019 criteria offer the highest diagnostic value, especially when applied to specific subtypes. The development of tailored diagnostic strategies based on disease phenotype, along with further validation in diverse populations, will be essential to improve the accuracy and utility of these criteria in clinical practice.

## Figures and Tables

**Table 1:** Patient clinical characteristics (*n* = 41).

	IgG4-RD (*n* = 30)	Non-IgG4-RD (*n* = 11)	*p* Value
Sex (male)	25 (83.3%)	4 (36.3%)	0.007
Race			0.47
- Caucasian	28 (96.6%)	10 (90.9%)	
- Asian	1 (3.4%)	1 (9.1%)	
Weight (kg), median (IQR)	83 (70.5–100.8)	84 (73.7–89.5)	0.665
Arterial hypertension	14 (46.7%)	4 (36.4%)	0.726
Dyslipidemia	14 (46.7%)	4 (36.4%)	0.726
Ischemic heart disease	6 (20%)	2 (18.2%)	1
Non-hematological neoplasia	1 (3.3%)	0 (0%)	1
Glandular involvement	4 (13.3%)	0 (0%)	1
- 1 set	2 (6.7%)		
- >2 sets	2 (6.7%)		
Pulmonary involvement			1
- Peri-bronchovascular and septal thickening	5 (16.7%)	1 (9.1%)	
- Paravertebral-like thorax soft tissue	0 (0%)	0 (0%)	
- Wall thickening of thoracic aorta	0 (0%)	0 (0%)	
Pancreatic involvement	10 (33.3%)	3 (27.27%)	0.557
- Lobulation loss	1 (3.3%)	0 (0%)	
- Low density capsule edging	2 (6.7%)	2 (18.2%)	
- Pancreatic and biliary injury	7 (23.3%)	1 (9.1%)	
Renal involvement	14 (46.7%)	1 (9.1%)	0.267
- Hypocomplementemia	3 (10%)	0 (0%)	
- Thickening of renal pelvis	3 (10%)	1 (9.1%)	
- Bilateral low-density renal cortex	8 (%)	0 (0%)	
Retroperitoneal involvement	17 (56.7%)	4 (36.4%)	0.593
- Wall thickening of abdominal aorta	7 (23.3%)	2 (18.2%)	
- Iliac or infrarenal aortic soft peripheral tissue	10 (33.3%)	2 (18.2%)	
Fever	3 (10%)	0 (0%)	0.551
Constitutional syndrome	3 (10%)	0 (0%)	0.551
Lower back pain	6 (20%)	0 (0%)	0.167
Hydronephrosis	3 (10%)	0 (0%)	0.551
Aneurysm	10 (33.3%)	3 (27.3%)	1
Hypophysitis	1 (3.3%)	1 (9.1%)	0.47
Pachymeningitis	1 (3.3%)	0 (0%)	0.268
Orbital pseudotumor	0 (0%)	0 (0%)	1
Uveal involvement	0 (0%)	0 (0%)	1
Sclera involvement	1 (9.1%)	0 (0%)	0.268
IgG, median (IQR)	1196 (979.2–1536.2)	1137 (1006.5–1250)	0.233
IgG4, median (IQR)	90.5 (40.8–154.5)	47.0 (33.5–86)	0.05
Plasmablasts, median (IQR)	993.0 (255.5–1628.2)	24 (0–101)	0.004

**Table 2:** Histological characteristics.

	IgG4-RD (*n* = 30)	Non-IgG4-RD (*n* = 11)	*p*-Value
Histology	9 (34.6%)	1 (9.09%)	1.000
Histological location - Liver - Adenopathy - Salivary gland - Abdominal mass - Pleura - Renal	1 (11.1%) 1 (11.1%) 2 (22.2%) 2 (22.2%) 1 (11.1%) 2 (22.2%)	1 (100.0%)	
Plasma cells	8 (88.9%)	1 (100.0%)	0.200
Phlebitis	2 (22.2%)	0 (0.0%)	1.000
Fibrosis	2 (22.2%)	0 (0.0%)	1.000

**Table 3:** Okazaki, Umehara, and ACR/EULAR 2019 diagnostic performance.

METRIC	OKAZAKI	UMEHARA	ACR/EULAR 2019
Confirmed IgG4-RD patients (*n* = 30)	18 (60%)	25 (83.33%)	20 (66.67%)
Sensitivity [95% CI]	60% [40.6–77.34%]	83.33% [65.28–94.36%]	66.67% [47.19–82.71%]
Specificity [95% CI]	100% [71.51–100%]	100% [71.51–100%]	100% [71.51–100%]
Accuracy [95% CI]	70.73% [54.46–83.87%]	87.8% [73.8–95.92%]	75.61% [59.7–87.64%]
Positive predictive value [95% CI]	100% [81.47–100%]	100% [86.28–100%]	100% [83.16–100%]
Negative predictive value [95% CI]	47.83% [26.82–69.41%]	68.75% [41.34–88.98%]	52.38% [29.78–74.29%]
Negative likelihood ratio [95% CI]	40% [25.81–62%]	16.67% [7.49–37.1%]	33.33% [20.1–55.29%]

Note: CI = confidence interval.

**Table 4:** IgG4 subtypes.

	SUBTYPE 1 Pancreato-Hepato-Biliary *n* = 8 (26.7%)	SUBTYPE 2 Retroperitoneal +/− Aortitis *n* = 19 (63.3%)	SUBTYPE 3 Head/Neck-Limited *n* = 1 (3.3%)	SUBTYPE 4 Mikulicz-Systemic Involvement *n* = 2 (6.7%)	*p* Value
Gender (male)	7 (87.5%)	16 (84.2%)	1 (100%)	1 (50%)	0.592
Race					1
- Caucasian	8 (100%)	18 (94.7%)	1 (100%)	2 (100%)	
- Asian	0 (0%)	1 (5.3%)	0 (0%)	0 (0%)	
Arterial hypertension	2 (25%)	12 (63.2%)	0 (0%)	0 (0%)	0.066
Dyslipidemia	3 (37.5%)	10 (52.6%)	0 (0%)	1 (50%)	0.83
Ischemic heart disease	0 (0%)	6 (31.6%)	0 (0%)	0 (0%)	0.321
Non-hematological neoplasia	0 (0%)	1 (5.3%)	0 (0%)	0 (0%)	1
Glandular involvement					1
- 1 set	0 (0%)	2 (10.5%)	0 (0%)	0 (0%)	
- >2 sets	1 (12.5%)	1 (5.3%)	0 (0%)	0 (0%)	
Pulmonary involvement					0.483
- Peri-bronchovascular and septal thickening	0 (0%)	5 (26.3%)	0 (0%)	0 (0%)	
- Paravertebral-like thorax soft tissue	0 (0%)	0 (0%)	0 (0%)	0 (0%)	
Pancreatic involvement					<0.001
- Lobulation loss	0 (0%)	1 (5.3%)	0 (0%)	0 (0%)	
- Low-density capsule edging	2 (25%)	0 (0%)	0 (0%)	0 (0%)	
- Pancreatic-biliary injury	6 (75%)	1 (5.3%)	0 (0%)	0 (0%)	
Renal involvement					0.914
- Hypocomplementemia	1 (12.5%)	2 (10.5%)	0 (0%)	0 (0%)	
- Thickening of renal pelvis	0 (0%)	3 (15.8%)	0 (0%)	0 (0%)	
- Bilateral low-density renal cortex	1 (12.5%)	6 (31.57%)	0 (0%)	1 (50%)	
Retroperitoneal involvement					0.001
- Wall thickening of abdominal aorta	0 (0%)	7 (36.8%)	0 (0%)	0 (0%)	
- Iliac and infrarenal aortic soft peripheral tissue	0 (0%)	10 (52.6%)	0 (0%)	0 (0%)	
Fever	0 (0%)	2 (10.5%)	0 (0%)	1 (50%)	0.293
Constitutional syndrome	0 (0%)	2 (10.5%)	0 (0%)	1 (50%)	0.293
Lower back pain	0 (0%)	6 (31.6%)	0 (0%)	0 (0%)	0.321
Hydronephrosis	0 (0%)	3 (15.8%)	0 (0%)	0 (0%)	0.663
Aneurysm	0 (0%)	9 (47.4%)	0 (0%)	1 (50%)	0.037
Hypophysitis	1 (12.5%)	0 (0%)	0 (0%)	0 (0%)	0.367
Okazaki 2009 criteria					0.902
- Not met	4 (50%)	7 (36.8%)	0 (0%)	1 (50%)	
- Met	4 (50%)	12 (63.2%)	1 (100%)	1 (50%)	
Umehara 2012 criteria					0.652
- Not met	0 (0%)	5 (26.3%)	0 (0%)	0 (0%)	
- Possible	4 (50%)	10 (52.6%)	1 (100%)	2 (100%)	
- Probable	1 (12.5%)	1 (5.3%)	0 (0%)	0 (0%)	
- Definitive	3 (37.5%)	3 (15.8%)	0 (0%)	0 (0%)	
ACR/EULAR 2019 criteria					0.653
- Not met	2 (25%)	6 (31.6%)	1 (100%)	1 (50%)	
- Met	6 (75%)	13 (68.4%)	0 (0%)	1 (50%)	
ACR/EULAR numeric	24.0	20.0	4.0	12.5	0.523
value, median (IQR)	(19.8–34.0)	(12.0–23.5)	(4.0–4.0)	(8.2–16.8)	
IgG, median (IQR)	1166.5	1180.0	1624.0	1355.5	0.390
	(853.2–1306.0)	(985.5–1542.0)	(1624.0–1624.0)	(1261.8–1449.2)	
IgG4, median (IQR)	72.0	84.0	153.0	297.0	0.45
	(39.8–132.8)	(43.5–180.0)	(153.0–153.0)	(207.0–387.0)	
Plasmablasts, median (IQR)	518.0	1005.0	9680.0	1176.5	0.179
	(0.0–862.2)	(288.0–1657.5)	(9680.0–9680.0)	(1145.8–1207.2)	

## Data Availability

The original contributions presented in the study are included in the article, further inquiries can be directed to the corresponding authors.

## Appendix A

### Okazaki Criteria (Proposed by the Japanese Research Committee for IgG4-Related Systemic Sclerosing Disease) 2009

1. Focal or diffuse enlargement or lesions in one or more organs.
2. Serum IgG4 concentrations > 135 mg/dL
3. Histopathology
  (a) Lymphocytic and plasmocytic infiltrate with fibrosis, without neutrophilic infiltrate.
  (b) IgG4 positive plasmocytic infiltrate greater than 10/cap or IgG4/IgG > 40% ratio of IgG4/IgG cells.
  (c) Storiform fibrosis
  (d) Obliterative phlebitis

The patient will be classified by meeting one of the following combinations of criteria:

- 1 + 2
- 1 + 3 (a + b)
- 2 + 3 (a + b)
- 3 (a + b + c + d)

## Appendix B

### Umehara Criteria 2011

1. Clinical examination showing characteristic diffuse/localized swelling or masses in one or more organs.
2. Hematologic examination showing elevated serum IgG4 concentrations (≥135 mg/dL).
3. Histopathologic examination showing:
  - Marked lymphocyte and plasmacyte infiltration and fibrosis.
  - IgG4-positive plasma cell infiltration: IgG4/IgG-positive cell ratio > 40% and >10 IgG4-positive plasma cells/high potency field (HPF).

The patient will be classified by meeting one of the following combinations of criteria:

Definitive: (1) + (2) + (3); Probable: (1) + (3); Possible: (1) + (2)

## Appendix C

### ACR/EULAR 2019 Criteria

#### Exclusion Criteria

Clinical

- fever: recurrent temperature > 38 °C without clinical signs of infection
- lack of response to glucocorticoids at doses of at least 40 mg/day of prednisone (approx. 0.6 mg/kg/day) for 4 weeks

Serologic

- leukopenia or thrombocytopenia without alternative explanation
- peripheral eosinophilia > 3000 mm^3^
- presence of ANCA antibodies
- presence of antinuclear antibodies anti-Ro, anti-La, anti-double-stranded DNA, anti-RNP, anti-Sm, anti-synthetase (e.g., anti-Jo1), antitopoisomerase III (Scl-70). This does not include antibodies of low specificity, such as rheumatoid factor, antinuclear antibodies (in screening), antimitochondrial antibodies, antimuscle smooth muscle antibodies, and antiphospholipid antibodies
- cryoglobulinemia.

Radiological

- radiological changes characteristic of neoplastic diseases or infections not sufficiently screened for
- rapid radiological progression
- anomalies in long bones compatible with Erdheim-Chester disease: multifocal osteosclerotic lesions in long bones
- splenomegaly: >14 cm without other explanations

Histopathologic

- cellular infiltrates that raise suspicion of a malignant neoplasm and have not been sufficiently analyzed
- markers related to an inflammatory myofibroblastic tumor
- evident neutrophilic inflammation
- necrotizing vasculitis
- evident necrosis (especially zonal necrosis)
- primary granulomatous inflammation
- characteristic signs of diseases related to macrophage/histiocyte disorders.

Histopathological

- cellular infiltrates that raise suspicion of a malignant neoplasm and have not been sufficiently analyzed
- markers related to an inflammatory myofibroblastic tumor
- evident neutrophilic inflammation
- necrotizing vasculitis
- evident necrosis (especially zonal necrosis)
- primary granulomatous inflammation
- characteristic signs of diseases related to macrophage/histiocyte disorders.

Exclusion of specific diseases

- multicentric Castleman’s disease
- Crohn’s disease
- ulcerative colitis
- Hashimoto’s thyroiditis

Inclusion criteria: a point count should be performed.

Histopathology	
-biopsy that does not provide relevant information	0
-dense lymphocytic infiltrate	4
-dense lymphocytic infiltrate and obliterative phlebitis	6
-dense lymphocytic infiltrate and glandular fibrosis with or without obliterating phlebitis	13
Immunostaining	0–16
Serum IgG4 concentration	
-normal or undetermined	0
-above normal, but <2× upper limit of normal (ULN)	4
-from 2× to 5× LSN	6
≥5× LSN	11
Bilateral involvement of lacrimal glands, parotid, sublingual and submaxillary salivary glands	
-unaffected	0
-affectation of 1 pair	6
-involvement of ≥2 pairs	14
Chest	
-undetermined or none of the aforementioned elements are present	0
-peri-bronchovascular and septal thickening	4
-paravertebral band-like soft tissue in the thorax	10
Pancreas and biliary tract	
-undetermined or none of the aforementioned elements are present	0
-diffuse enlargement of the pancreas (loss of lobulation)	8
-diffuse enlargement of the pancreas and low-density capsule rim	11
-involvement of the pancreas (one of the above) and bile ducts	19
Kidney	
-undetermined or none of the aforementioned elements are present	0
-hypocomplementemia	6
-thickening of the renal pelvis/soft tissue	8
– bilateral areas of low density in the renal cortex	10
Retroperitoneum	
-undetermined or none of the aforementioned elements are present	0
-diffuse thickening of the wall of the abdominal aorta	4
-peripheral or anterolateral soft tissue around the infrarenal aorta or iliac arteries	8

IgG4-related disease classification criteria are met if the entry criterion is met, there are no exclusion criteria, and the total score is ≥20.
